# Psychedelic use and psychiatric risks

**DOI:** 10.1007/s00213-023-06478-5

**Published:** 2023-10-24

**Authors:** Otto Simonsson, Simon B. Goldberg, Richard Chambers, Walter Osika, Charlotta Simonsson, Peter S. Hendricks

**Affiliations:** 1 Center for Psychiatry Research, Department of Clinical Neuroscience, Karolinska Institute, Stockholm, Sweden; 2 Center for Social Sustainability, Department of Neurobiology, Care Sciences and Society, Karolinska Institute, Stockholm, Sweden; 3 Department of Sociology, University of Oxford, Oxford, UK; 4 Department of Counseling Psychology, University of Wisconsin – Madison, Madison, WI USA; 5 Monash Centre for Consciousness & Contemplative Studies, Monash University, Melbourne, Australia; 6 Faculty of Medicine and Health Sciences, Linköping University, Linköping, Sweden; 7 Department of Psychiatry and Behavioral Neurobiology, School of Medicine, University of Alabama at Birmingham, Birmingham, AL USA

**Keywords:** Psychedelics, Risks, HPPD, Psychotic, Visual

## Abstract

**Rationale:**

Research on psychedelics has recently shown promising results in the treatment of various psychiatric disorders, but relatively little remains known about the psychiatric risks associated with naturalistic use of psychedelics.

**Objective:**

The objective of the current study was to investigate associations between naturalistic psychedelic use and psychiatric risks.

**Methods:**

Using a sample representative of the US adult population with regard to sex, age, and ethnicity (*N*=2822), this study investigated associations between lifetime naturalistic psychedelic use, lifetime unusual visual experiences, and past 2-week psychotic symptoms.

**Results:**

Among respondents who reported lifetime psychedelic use (*n*=613), 1.3% reported having been told by a doctor or other medical professional that they had hallucinogen persisting perception disorder. In covariate-adjusted linear regression models, lifetime psychedelic use was associated with more unusual visual experiences at any point across the lifetime, but no association was observed between lifetime psychedelic use and past 2-week psychotic symptoms. There was an interaction between lifetime psychedelic use and family (but not personal) history of psychotic or bipolar disorders on past 2-week psychotic symptoms such that psychotic symptoms were highest among respondents who reported lifetime psychedelic use and a family history of psychotic or bipolar disorders and lowest among those who reported lifetime psychedelic use and no family history of psychotic or bipolar disorders.

**Conclusions:**

Although the results in this study should be interpreted with caution, the findings suggest that lifetime naturalistic use of psychedelics might be associated with more unusual visual experiences across the lifetime, as well as more psychotic symptoms in the past 2 weeks for individuals with a family history of psychotic or bipolar disorders and the reverse for those without such a family history. Future research should distinguish between different psychotic and bipolar disorders and should also utilize other research designs (e.g., longitudinal) and variables (e.g., polygenic risk scores) to better understand potential cause-and-effect relationships.

**Supplementary Information:**

The online version contains supplementary material available at 10.1007/s00213-023-06478-5.

Research on psychedelics such as psilocybin and lysergic acid diethylamide (LSD) has recently shown promising results in the treatment of various psychiatric disorders (Andersen et al. 2021; Galvão-Coelho et al. 2021). For example, in a recent double-blind randomized, controlled trial with patients who had been diagnosed with moderate-to-severe major depressive disorder, psilocybin-assisted therapy was at least as effective as an active control condition (escitalopram) in reducing depressive symptoms (Carhart-Harris et al. 2021; see also, Goodwin et al. 2022; von Rotz et al. 2023). The evidence to date suggests that psychedelics generally have a favorable safety profile (Roscoe and Lozy 2022), but psychedelic trials are characterized by strict exclusion criteria and relatively little remains known about the range of possible adverse events (Schlag et al. 2022). It is therefore important to further investigate potential risks associated with psychedelic use, especially among populations that are typically excluded from participation in psychedelic trials (e.g., personal or family history of psychotic or bipolar disorders; Johnson et al. 2008).

One potential risk associated with psychedelic use is visual hallucinations or flashback-type experiences (e.g., halos around objects, macropsia, micropsia) occurring after the acute pharmacological effects have subsided (Baggott et al. 2011; Müller et al. 2022; but see Krebs and Johansen 2013). Such experiences can be diagnosed as hallucinogen persisting perception disorder (HPPD; Halpern and Pope Jr 2003) if (1) the visual phenomena persist and cause significant distress or impairment in daily functioning and (2) other medical or psychiatric conditions can be ruled out (American Psychiatric Association 2013; see Halpern et al. 2016 for proposed HPPD subtypes). Yet, the evidence on the prevalence and predictors of unusual visual experiences and HPPD-like symptoms remains relatively limited.

Another concern is that psychedelic use might in rare cases provoke the onset of prolonged psychosis (Strassman 1984), but the evidence has been mixed so far. For example, having used psychedelics five or more times in the past was associated with lifetime experience of two or more psychotic symptoms, in a representative community sample of adolescents and young adults in Germany (Kuzenko et al. 2011). Another study, by contrast, found no association between lifetime psychedelic use and two or more psychotic symptoms in the past year, in a nationally representative sample of adults in the USA (Krebs and Johansen 2013; see also Lebedev et al. 2021). The differences in results across studies may be explained by the heterogeneous research designs, but these studies also did not investigate whether the association between psychedelic use and psychotic symptoms was stronger in populations with a genetic risk for certain psychiatric disorders (e.g., psychotic or bipolar disorders), which could provide insight into the potential risks associated with psychedelic use for these populations.

It is not ethically tenable to experimentally test if, for whom, and under what circumstances psychedelic use may have potentially harmful effects (e.g., HPPD-like symptoms, psychotic symptoms), which highlights the need for epidemiological research on potential psychiatric risks associated with the use of psychedelics. Using a sample representative of the US adult population with regard to sex, age, and ethnicity (*N*=2822), the objective of the current study was to investigate associations between naturalistic psychedelic use, unusual visual experiences, and psychotic symptoms.

## Methods

### Participants and procedure

Using linear multiple regression in GPower 3.1 (Faul et al. 2009), it was determined that a sample size of 395 psychedelic users would achieve 80% power to detect a small effect size (Cohen’s f2 of .02) with an alpha of .05. Based on recent data on the prevalence of lifetime psychedelic use in the US adult population (~14%; Simonsson et al. 2021), we estimated approximately 2800 participants would be necessary to obtain 395 psychedelic users in the sample. We aimed to recruit 2800 participants in total.

Participants were current residents of the USA (≥18 years old) and were recruited on Prolific Academic (https://app.prolific.co). The sample (*N*=2822) was collected in October (1st–9th) 2021 and was stratified across three demographic characteristics—sex, age, and ethnicity—to reflect the demographic distribution of the US adult population. The participants were asked about demographic characteristics, substance use (including psychedelics), unusual visual experiences, and psychotic symptoms. Study completion resulted in $2.20 payment and study procedures were determined to be exempt by the Institutional Review Board at the University of Wisconsin–Madison. The data and Stata syntax are available at 10.6084/m9.figshare.24316432.v1.

## Measures

### Demographics and substance use

All respondents were asked to report age in years, gender, ethnoracial identity, educational attainment, annual household income, marital status, engagement in risky behavior, and lifetime use of cocaine, sedatives, pain relievers, marijuana, phencyclidine (PCP), 3,4-methylenedioxymethamphetamine (MDMA/ecstasy), and inhalants.

### Lifetime psychedelic use

All respondents were asked to report which, if any, of the following psychedelics they had ever used: ayahuasca, N,N-Dimethyltryptamine (DMT), LSD, mescaline, peyote, or San Pedro, or psilocybin. Respondents who reported that they had used any of these substances were coded as 1, whereas those indicating that they had never used any of these substances were coded as 0.

### Unusual visual experiences

All respondents completed the 9-item unusual visual experiences scale (Baggott et al. 2011), which asks respondents to report if they have ever had any of the listed unusual visual experiences (e.g., “Stationary things appear to move, breathe, grow, or shrink”), excluding times when they were intoxicated or had used drugs in the past 3 days and times when they were in trance, falling asleep, waking up, or had not been sleeping for a long time. Internal consistency in the current sample was adequate (alpha = .80). The total score was calculated by summing across items. Similar to Baggott et al. (2011), respondents who endorsed any of the first seven listed unusual visual experiences were asked about the frequency of those experiences (very rarely, rarely, occasionally, very frequently, constantly) and also whether these unusual visual experiences overall had been so troublesome or had made social, work, school, or other activities so difficult that they had considered or sought professional treatment (treatment not considered, treatment considered, treatment sought).

### Hallucinogen persisting perception disorder (HPPD)

All respondents were asked whether a doctor or other medical professional had ever told them that they had HPPD (yes = 1, no = 0).

### Psychotic symptoms

All respondents completed the 6-item psychotic ideation subscale of the Psychiatric Diagnostic Screening Questionnaire (PDSQ; Zimmerman and Mattia 2001), which asks respondents to report psychotic symptoms during the past 2 weeks (e.g., “During the past two weeks, did you think that you had special powers other people didn’t have?”). Internal consistency in the current sample was adequate (alpha = .69). The total score was calculated by summing across items.

### Personal and family history of psychotic disorders, or bipolar disorders I or II

All respondents were asked to report whether they had a current or past history of any psychotic disorders or bipolar I or II disorders, as well as whether they had a first- or second-degree relative with any psychotic disorders or bipolar I or II disorders. For personal history, respondents who reported that they had a current or past history of any psychotic or bipolar disorders were coded as 1, whereas those indicating that they did not have a current or past history of any psychotic or bipolar disorders were coded as 0. For family history, respondents who reported they had a first- or second-degree relative with any psychotic or bipolar disorders were coded as 1, whereas those indicating that they did not have a first- or second-degree relative with any psychotic or bipolar disorders were coded as 0.

### Statistical analyses

We used Pearson’s chi-squared tests (for categorical variables) and *t*-tests (for continuous variables) to examine unadjusted differences between the two groups (users, non-users). Two separate multiple linear regression models were then used to evaluate associations of lifetime psychedelic use (the independent variable in both models) with unusual visual experiences (the dependent variable in model 1) and psychotic symptoms (the dependent variable in model 2). A third multiple linear regression model (model 3) evaluated the interaction between lifetime psychedelic use and personal history of psychotic or bipolar disorders on psychotic symptoms. The purpose of this model was to determine whether psychedelic use might aggravate psychotic symptoms, although the temporal relationship between age of first psychedelic use and age of diagnosis was not investigated. A fourth multiple linear regression model (model 4) evaluated the interaction between lifetime psychedelic use and family history of psychotic or bipolar disorders on psychotic symptoms. The purpose of the fourth model was to determine whether psychedelic use might be more strongly associated with psychotic symptoms among those genetically predisposed to psychotic or bipolar disorders. As sensitivity analyses, we also ran all four analyses using multiple logistic regression models with the dependent variables dichotomized (i.e., one or more unusual visual experiences = 1, no unusual visual experiences = 0; one or more psychotic symptoms = 1, no psychotic symptoms = 0).

In all models, we controlled for broadly the same covariates that were used in the only prior study that has evaluated the associations between lifetime psychedelic use, unusual visual experiences, and psychotic symptoms in a sample representative of the US adult population (Krebs and Johansen 2013): age in years, gender, ethnoracial identity, educational attainment, annual household income, marital status, engagement in risky behavior, and lifetime use of cocaine, sedatives, pain relievers, marijuana, phencyclidine (PCP), 3,4-methylenedioxymethamphetamine (MDMA/ecstasy), and inhalants (each drug use variable entered as a separate covariate). Due to a data collection error, not all covariates in Krebs and Johansen (2013) were included in this study (e.g., lifetime exposure to an extremely stressful event). For all analyses, *p*-values are reported with 3 decimal places, allowing the reader to estimate any *p*-value corrections of the reader’s choosing.

## Results

### Descriptive statistics and sample characteristics

Tables 1 and 2 show descriptive statistics and sample characteristics. As seen in Table 1, respondents who reported lifetime psychedelic use had significantly higher scores on unusual visual experiences across the lifetime than those who did not report previous psychedelic use, but there were no differences in past 2-week psychotic symptoms between those who reported lifetime psychedelic use and those who did not (see Supplemental Table 1 for additional descriptive statistics). As shown in Table 2, personal and family histories of psychotic or bipolar disorders were significantly more common among psychedelic users. Notably, having been told by a doctor or other medical professional that they had HPPD was significantly more common among psychedelic users (1.3% versus 0.3% among non-users), but no differences were observed across groups in frequency or treatment of unusual visual experiences.

**Table 1:** Descriptive statistics of psychotic symptoms and unusual visual experiences

	Users (*n*=613)	Non-users (*n*=2209)	
	Mean (SD)	Mean (SD)	*p*
Unusual visual experiences	1.82 (1.89)	0.75 (1.51)	<.001
Psychotic symptoms	0.30 (0.81)	0.33 (0.86)	.396

Mean, the mean number of unusual visual experiences or psychotic symptoms in the group; *SD*, standard deviation; *t*-tests were used to compare the means across groups (users, non-users)

**Table 2:** Sample characteristics of lifetime psychedelic users versus non-users

	Users (*n*=613)	Non-users (*n*=2209)	
	*n* (%)	*n* (%)	*p*
18–25 years old	81 (13.1)	379 (17.2)	.019
Male	332 (54.2)	1,033 (46.8)	.001
Non-Hispanic White	523 (85.3)	1577 (71.4)	<.001
Bachelor’s degree or higher	301 (49.1)	1392 (63.0)	<.001
Annual household income of ≥US$75,000	202 (33.0)	900 (40.7)	<.001
Single or never married	172 (28.1)	616 (27.9)	.933
Never risk-taking	47 (7.7)	293 (13.3)	<.001
Lifetime cocaine use	425 (69.3)	226 (10.2)	<.001
Lifetime sedative use	323 (52.7)	518 (23.5)	<.001
Lifetime pain reliever use	510 (83.2)	1357 (61.4)	<.001
Lifetime marijuana use	595 (97.1)	1208 (54.7)	<.001
Lifetime PCP use	87 (14.2)	31 (1.4)	<.001
Lifetime MDMA/ecstasy use	251 (41.0)	102 (4.6)	<.001
Lifetime inhalants	244 (39.8)	295 (13.4)	<.001
Personal history of psychotic or bipolar disorders	72 (11.8)	83 (3.8)	<.001
Family history of psychotic or bipolar disorders	190 (31.0)	411 (18.6)	<.001
HPPD reported by doctor or other medical professional	8 (1.3)	6 (0.3)	.001
	Users (*n*=205)	Non-users (*n*=530)	
Frequency of unusual visual experiences			.772
Very rarely	57 (27.8)	153 (28.9)	
Rarely	62 (30.2)	171 (32.3)	
Occasionally	62 (30.2)	159 (30.0)	
Very frequently	21 (10.2)	43 (8.1)	
Constantly	3 (1.5)	4 (0.8)	
Treatment of unusual visual experiences			.194
Treatment not considered	156 (76.1)	369 (69.6)	
Treatment considered	29 (14.2)	102 (19.3)	
Treatment sought	20 (9.8)	59 (11.1)	

*n* refers to the number of respondents in the respective columns (users, non-users) who endorsed each item; percentages are reported within brackets. Respondents who endorsed any of the first seven listed unusual visual experiences (users, *n* = 205; non-users, *n* = 530) were asked about the frequency and treatment of those experiences. All percentages were rounded to the nearest 0.1%; cumulative percentages may not add to 100.0. Pearson’s chi-squared tests were used to examine the characteristics of lifetime psychedelic users versus non-users

### Covariate-adjusted regression models

Table 3 presents results from four separate multiple linear regression models on the associations of lifetime psychedelic use with unusual visual experiences and psychotic symptoms. While lifetime psychedelic use was associated with more unusual visual experiences at any point across the lifetime, no association was observed between lifetime psychedelic use and recent psychotic symptoms. There was an interaction between lifetime psychedelic use and family (but not personal) history of psychotic or bipolar disorders on psychotic symptoms such that psychotic symptoms were highest​ among respondents who reported lifetime psychedelic use and a family history of psychotic or bipolar disorders and lowest​ among those who reported lifetime psychedelic use and no family history of psychotic or bipolar disorders (see Supplemental Table 2 for adjusted means). Sensitivity analyses showed broadly the same results (see Supplemental Table 3 for results).

**Table 3:** Multiple linear regression model estimates

	B (CI 95%)	*p*	*R*^2^
Model 1			.09
Lifetime psychedelic use predicting lifetime unusual visual experiences	0.31 (0.13–0.49)	.001	
Model 2			.13
Lifetime psychedelic use predicting past 2-week psychotic symptoms	−0.04 (−0.13–0.06)	.445	
Model 3			.13
*Main effects predicting past 2-week psychotic symptoms*			
Lifetime psychedelic use	−0.05 (−0.15−0.05)	.318	
Personal history of psychotic or bipolar disorders	0.39 (0.21–0.57)	<.001	
*Interaction effects predicting past 2-week psychotic symptoms*			
Lifetime psychedelic use X personal history of psychotic or bipolar disorders	−0.02 (−0.28–0.24)	.888	
Model 4			.13
*Main effects predicting past 2-week psychotic symptoms*			
Lifetime psychedelic use	−0.10 (−0.21–0.00)	.052	
Family history of psychotic or bipolar disorders	0.05 (−0.04–0.14)	.281	
*Interaction effects predicting past 2-week psychotic symptoms*			
Lifetime psychedelic use X family history of psychotic or bipolar disorders	0.21 (0.05–0.38)	.010	

*B*, unstandardized beta; *R*^2^, R-squared. Four separate multiple linear regression models were used to evaluate associations. All regression models controlled for age in years, gender, ethnoracial identity, educational attainment, annual household income, marital status, engagement in risky behavior, lifetime use of cocaine, sedatives, pain relievers, marijuana, phencyclidine (PCP), 3,4-methylenedioxymethamphetamine (MDMA/ecstasy), and inhalants

## Discussion

The present study investigated the associations between lifetime naturalistic psychedelic use, lifetime unusual visual experiences, and past 2-week psychotic symptoms in a sample representative of the US adult population with regard to sex, age, and ethnicity. Although the results in this study should be interpreted with caution, the findings suggest that lifetime use of psychedelics might be associated with more unusual visual experiences across the lifetime, as well as more psychotic symptoms in the past 2 weeks for individuals with a family history of psychotic or bipolar disorders and the reverse for those without such a family history.

Lifetime psychedelic use was associated with unusual visual experiences at any point across the lifetime. While such experiences may not have been functionally impairing, the results showed that 1.3% of respondents who reported lifetime psychedelic use had been told by a doctor or other medical professional that they had HPPD. Previous research on lifetime users of psychedelics and other substances (e.g., cannabis, ketamine, MDMA) found that at least 1.7% of respondents had a clear temporal relationship between drug use and onset of HPPD-like symptoms (Baggott et al. 2011), which is a statistic that broadly corresponds with the HPPD prevalence reported in this study. Given that potential risk factors (e.g., genetic, extra-pharmacological) of these types of experiences are not yet well-understood, future studies should use longitudinal research designs to better understand if, for whom, and under what circumstances psychedelic use might lead to unusual visual experiences.

Lifetime psychedelic use was not directly associated with recent psychotic symptoms, but there was an interaction between lifetime psychedelic use and family history of psychotic or bipolar disorders on psychotic symptoms such that psychotic symptoms were highest​ among respondents who reported lifetime psychedelic use and a family history of psychotic or bipolar disorders and lowest​ among those who reported lifetime psychedelic use and no family history of psychotic or bipolar disorders. These findings suggest that there may be a genetic predisposition that puts certain individuals at risk of psychotic symptoms following use of psychedelics, which corresponds with the leading guidelines for psychedelic research (Johnson et al. 2008). Previous research suggests that psychotic disorders such as schizophrenia are genetically related to bipolar disorders (Ruderfer et al. 2018), but it is possible that the effects of psychedelic use on psychotic symptoms might differ between these disorders. It is also possible that psychedelics can induce a manic switch and put certain individuals at risk of mania (potentially with psychotic features; Gard et al. 2021), which highlights the need for future research to investigate psychotic and bipolar disorders separately and also to examine both psychotic and manic symptoms.

There are many features of the study design that should be considered when interpreting the results. First, causality cannot be inferred due to the cross-sectional research design. Any findings suggesting psychedelic use elicits unusual visual experiences or psychotic symptoms could also be interpreted to mean that unusual visual experiences or psychotic symptoms elicit psychedelic use. Consistent with this interpretation, family history of psychotic or bipolar disorders (i.e., a variable that could not be affected by lifetime psychedelic use) was more commonly reported among those who reported lifetime use of psychedelics. Second, even though the sample was stratified across sex, age, and ethnicity to reflect the demographic distribution of the US adult population, it may not have been representative on other relevant variables such as income or educational attainment. It is also possible that associations in the covariate-adjusted models were influenced by other variables not included in the survey that are relevant to unusual visual experiences and psychotic symptoms (e.g., amphetamine use). Fourth, the survey item related to HPPD did not specify whether it concerned HPPD type I or II. Given that the estimated prevalence rate of HPPD type II among hallucinogen users is extremely low (Halpern et al. 2016), it is possible that those respondents who reported HPPD diagnosis by a doctor or other medical professional had received a HPPD type I diagnosis. It should be noted, however, that these may have been inaccurate diagnoses. For example, it is possible that distressing symptoms associated with psychedelic use were labeled HPPD as a diagnostic category necessary for third party reimbursement. Fifth, the questionnaires in this study used the same phrases that were used in the original questionnaires, which resulted in different time frames for unusual visual experiences (at any point across the lifetime) and psychotic symptoms (past 2 weeks). This suggests that the reported associations of psychedelic use with unusual visual experiences and psychotic symptoms may not be comparable. Sixth, respondents were asked whether they had a current or past history of a psychotic or bipolar disorder and also whether they had a first- or second-degree relative with a psychotic or bipolar disorder, which corresponds with criteria that would typically lead to exclusion from participation in a clinical trial with psychedelics. The respondents were not, however, asked to specify whether they had either a personal or family history of either psychotic disorders or bipolar disorders per se (or subtypes thereof), which would have been useful in determining the associations of each unique disorder. Future research should use longitudinal research designs to investigate interaction effects between psychedelic use and potentially relevant psychiatric histories (e.g., anxiety disorders) on unusual visual experiences. It would also be important to investigate interaction effects between psychedelic use and more specific psychiatric histories (e.g., schizophrenia, brief psychotic disorder, bipolar I disorder, bipolar II disorder) on psychotic and manic symptoms.

## Conclusions

Despite the limitations of this study, the current results suggest that lifetime naturalistic use of psychedelics might be associated with more unusual visual experiences across the lifetime, as well as more psychotic symptoms in the past 2 weeks for individuals with a family history of psychotic or bipolar disorders and the reverse for those without such a family history. Future research should distinguish between different psychotic and bipolar disorders and specify subtypes of these mental health conditions and should also utilize other research designs (e.g., longitudinal) and variables (e.g., polygenic risk scores) to better understand potential cause-and-effect relationships.

## Supplementary information

ESM 1 (DOCX 107 kb)

## Data Availability

The data and Stata syntax are available at 10.6084/m9.figshare.24316432.v1.
