# Supplementary material for: Psychedelic use and psychiatric risks
Source: Psychopharmacology (Berl). 2023 Oct 24;242(7):1577–83. doi: 10.1007/s00213-023-06478-5 (PMC11039563; doi:10.1007/s00213-023-06478-5)
Supplement: Supplementary file 1 — (DOCX 107 kb) [file 213_2023_6478_MOESM1_ESM.docx]

**Psychedelic Use and Psychiatric Risks**

Authors: Otto Simonsson^1, 2, 3^, Simon Goldberg^4^, Richard Chambers^5^, Walter Osika^1, 2^, Charlotta Simonsson^6^, Peter S. Hendricks^7^

^1^Center for Psychiatry Research, Department of Clinical Neuroscience, Karolinska Institute, Stockholm, Sweden

^2^Center for Social Sustainability, Department of Neurobiology, Care Sciences and Society, Karolinska Institute, Stockholm, Sweden

^3^Department of Sociology, University of Oxford, Oxford, UK

^4^Department of Counseling Psychology, University of Wisconsin - Madison, Madison, WI, USA

^5^Monash Centre for Consciousness & Contemplative Studies, Monash University, Melbourne, Australia

^6^Faculty of Medicine and Health Sciences, Linköping University, Linköping, Sweden

^7^Department of Psychiatry and Behavioral Neurobiology, School of Medicine, University of Alabama at Birmingham, Birmingham, AL, USA

Corresponding Author Contact Details

Otto Simonsson, otto.simonsson@ki.se

ORCID numbers

Otto Simonsson: 0000-0003-4197-7566

Simon B. Goldberg: 0000-0002-6888-0126

Walter Osika: 0000-0002-1583-7319

***Disclosure statement***

PSH has been in paid advisory relationships with the following organizations regarding the development of psychedelics and related compounds: Bright Minds Biosciences Ltd., Eleusis Benefit Corporation, Journey Colab Corporation, Reset Pharmaceuticals Inc., and Silo Pharma. OS and RC were co-founders of Eudelics AB.

***Funding***

OS was supported by Osmond Foundation, Ekhaga Foundation and Olle Engkvist Foundation. SG was supported by a grant (K23AT010879) from the National Center for Complementary and Integrative Health. Support for this research was also provided by FORMAS, Swedish Research Council for Sustainable Development (Grant number: FR-2018/0006), Three Springs Foundation through the Monash Centre for Consciousness & Contemplative Studies, the University of Alabama at Birmingham School of Public Health, and the University of Wisconsin - Madison Office of the Vice Chancellor for Research and Graduate Education with funding from the Wisconsin Alumni Research Foundation and with funding from the Wisconsin Center for Education Research.

***Ethical approval***

All procedures performed involving human participants were in accordance with the ethical standards of the 1964 Helsinki declaration and its later amendments or comparable ethical standards. The study was deemed to be exempt by the Internal Review Board (IRB) at UW-Madison.

***Informed consent***

Informed consent was obtained from all individual participants included in the studies.

***Author Contributions***

OS conceptualized and designed the study, with input from SG and PSH. OS analyzed the data and wrote the manuscript. All drafts received comments and input from SG, PSH, RC, and WO.

***Abstract***

*Rationale*

Research on psychedelics has recently shown promising results in the treatment of various psychiatric disorders, but relatively little remains known about the psychiatric risks associated with naturalistic use of psychedelics.

*Objective*

The objective of the current study was to investigate associations between naturalistic psychedelic use and psychiatric risks.

*Methods*

Using a sample representative of the US adult population with regard to sex, age, and ethnicity (N=2,822), this study investigated associations between lifetime naturalistic psychedelic use, lifetime unusual visual experiences, and past two-week psychotic symptoms.

*Results*

Among respondents who reported lifetime psychedelic use (n=613), 1.3 percent reported having been told by a doctor or other medical professional that they had hallucinogen persisting perception disorder. In covariate-adjusted linear regression models, lifetime psychedelic use was associated with more unusual visual experiences at any point across the lifetime, but no association was observed between lifetime psychedelic use and past two-week psychotic symptoms. There was an interaction between lifetime psychedelic use and family (but not personal) history of psychotic or bipolar disorders on past two-week psychotic symptoms such that psychotic symptoms were highest​ among respondents who reported lifetime psychedelic use and a family history of psychotic or bipolar disorders, and lowest​ among those who reported lifetime psychedelic use and no family history of psychotic or bipolar disorders.

*Conclusions*

Although the results in this study should be interpreted with caution, the findings suggest that lifetime naturalistic use of psychedelics might be associated with more unusual visual experiences across the lifetime, as well as more psychotic symptoms in the past two weeks for individuals with a family history of psychotic or bipolar disorders and the reverse for those without such a family history. Future research should distinguish between different psychotic and bipolar disorders and should also utilize other research designs (e.g., longitudinal) and variables (e.g., polygenic risk scores) to better understand potential cause-and-effect relationships.

Research on psychedelics such as psilocybin and lysergic acid diethylamide (LSD) has recently shown promising results in the treatment of various psychiatric disorders (Andersen et al., 2021; Galvão-Coelho et al., 2021). For example, in a recent double-blind randomized, controlled trial with patients who had been diagnosed with moderate-to-severe major depressive disorder, psilocybin-assisted therapy was at least as effective as an active control condition (escitalopram) in reducing depressive symptoms (Carhart-Harris et al., 2021; see also, Goodwin et al., 2022; von Rotz et al., 2023). The evidence to date suggests that psychedelics generally have a favorable safety profile (Roscoe & Lozy, 2022), but psychedelic trials are characterized by strict exclusion criteria and relatively little remains known about the range of possible adverse events (Schlag et al., 2022). It is therefore important to further investigate potential risks associated with psychedelic use, especially among populations that are typically excluded from participation in psychedelic trials (e.g., personal or family history of psychotic or bipolar disorders; Johnson et al., 2008).

One potential risk associated with psychedelic use is visual hallucinations or flashback-type experiences (e.g., halos around objects, macropsia, micropsia) occurring after the acute pharmacological effects have subsided (Baggott et al., 2011; Müller et al., 2022; but see Krebs & Johansen, 2013). Such experiences can be diagnosed as hallucinogen persisting perception disorder (HPPD; Halpern & Pope, 2003) if: (1) the visual phenomena persist and cause significant distress or impairment in daily functioning; and (2) other medical or psychiatric conditions can be ruled out (American Psychiatric Association, 2013; see Halpern et al., 2018 for proposed HPPD subtypes). Yet, the evidence on the prevalence and predictors of unusual visual experiences and HPPD-like symptoms remains relatively limited.

Another concern is that psychedelic use might in rare cases provoke the onset of prolonged psychosis (Strassman, 1984), but the evidence has been mixed so far. For example, having used psychedelics five or more times in the past was associated with lifetime experience of two or more psychotic symptoms, in a representative community sample of adolescents and young adults in Germany (Kuzenko et al., 2011). Another study, by contrast, found no association between lifetime psychedelic use and two or more psychotic symptoms in the past year, in a nationally representative sample of adults in the United States (Krebs & Johansen, 2013; see also Lebedev et al., 2021). The differences in results across studies may be explained by the heterogeneous research designs, but these studies also did not investigate whether the association between psychedelic use and psychotic symptoms was stronger in populations with a genetic risk for certain psychiatric disorders (e.g., psychotic or bipolar disorders), which could provide insight into the potential risks associated with psychedelic use for these populations.

It is not ethically tenable to experimentally test if, for whom, and under what circumstances psychedelic use may have potentially harmful effects (e.g., HPPD-like symptoms, psychotic symptoms), which highlights the need for epidemiological research on potential psychiatric risks associated with the use of psychedelics. Using a sample representative of the US adult population with regard to sex, age, and ethnicity (N=2,822), the objective of the current study was to investigate associations between naturalistic psychedelic use, unusual visual experiences, and psychotic symptoms.

**Methods**

**Participants and Procedure**

Using linear multiple regression in GPower 3.1 (Faul et al., 2009), it was determined that a sample size of 395 psychedelic users would achieve 80% power to detect a small effect size (Cohen’s f2 of .02) with an alpha of .05. Based on recent data on the prevalence of lifetime psychedelic use in the US adult population (~14%; Simonsson et al., 2021), we estimated approximately 2800 participants would be necessary to obtain 395 psychedelic users in the sample. We aimed to recruit 2800 participants in total.

Participants were current residents of the United States of America (≥18 years old) and were recruited on Prolific Academic (<https://app.prolific.co>). The sample (N=2,822) was collected in October (1^st^ – 9^th^) 2021 and was stratified across three demographic characteristics – sex, age and ethnicity – to reflect the demographic distribution of the US adult population. The participants were asked about demographic characteristics, substance use (including psychedelics), unusual visual experiences, and psychotic symptoms. Study completion resulted in $2.20 payment and study procedures were determined to be exempt by the Institutional Review Board at the University of Wisconsin – Madison. The data and Stata syntax are available at [Insert link here upon acceptance].

**Measures**

***Demographics and substance use***

All respondents were asked to report age in years, gender, ethnoracial identity, educational attainment, annual household income, marital status, engagement in risky behavior, and lifetime use of cocaine, sedatives, pain relievers, marijuana, phencyclidine (PCP), 3,4-methylenedioxymethamphetamine (MDMA/ecstasy), and inhalants.

***Lifetime psychedelic use***

All respondents were asked to report which, if any, of the following psychedelics they had ever used: ayahuasca, N,N-Dimethyltryptamine (DMT), LSD, mescaline, peyote, or San Pedro, or psilocybin. Respondents who reported that they had used any of these substances were coded as 1, whereas those indicating that they had never used any of these substances were coded as 0.

***Unusual visual experiences***

All respondents completed the 9-item unusual visual experiences scale (Baggott et al., 2011), which asks respondents to report if they have ever had any of the listed unusual visual experiences (e.g., “Stationary things appear to move, breathe, grow, or shrink”), excluding times when they were intoxicated or had used drugs in the past three days and times when they were in trance, falling asleep, waking up, or had not been sleeping for a long time. Internal consistency in the current sample was adequate (alpha = .80). The total score was calculated by summing across items. Similar to Baggott and colleagues (2011), respondents who endorsed any of the first seven listed unusual visual experiences were asked about the frequency of those experiences (very rarely, rarely, occasionally, very frequently, constantly) and also whether these unusual visual experiences overall had been so troublesome, or had made social, work, school, or other activities so difficult that they had considered or sought professional treatment (treatment not considered, treatment considered, treatment sought).

***Hallucinogen persisting perception disorder (HPPD)***

All respondents were asked whether a doctor or other medical professional had ever told them that they had HPPD (yes = 1, no = 0).

***Psychotic symptoms***

All respondents completed the 6-item psychotic ideation subscale of the Psychiatric Diagnostic Screening Questionnaire (PDSQ; Zimmerman & Mattia, 2001), which asks respondents to report psychotic symptoms during the past two weeks (e.g., “During the past two weeks, did you think that you had special powers other people didn’t have?”). Internal consistency in the current sample was adequate (alpha = .69). The total score was calculated by summing across items.

***Personal and family history of psychotic disorders, or bipolar disorders I or II***

All respondents were asked to report whether they had a current or past history of any psychotic disorders or bipolar I or II disorders, as well as whether they had a first or second-degree relative with any psychotic disorders or bipolar I or II disorders. For personal history, respondents who reported that they had a current or past history of any psychotic or bipolar disorders were coded as 1, whereas those indicating that they did not have a current or past history of any psychotic or bipolar disorders were coded as 0. For family history, respondents who reported they had a first or second-degree relative with any psychotic or bipolar disorders were coded as 1, whereas those indicating that they did not have a first or second-degree relative with any psychotic or bipolar disorder were coded as 0.

***Statistical analyses***

We used Pearson's chi-squared tests (for categorical variables) and t-tests (for continuous variables) to examine unadjusted differences between the two groups (users, non-users). Two separate multiple linear regression models were then used to evaluate associations of lifetime psychedelic use (the independent variable in both models) with unusual visual experiences (the dependent variable in Model 1) and psychotic symptoms (the dependent variable in Model 2). A third multiple linear regression model (Model 3) evaluated the interaction between lifetime psychedelic use and personal history of psychotic or bipolar disorders on psychotic symptoms. The purpose of this model was to determine whether psychedelic use might aggravate psychotic symptoms, although the temporal relationship between age of first psychedelic use and age of diagnosis was not investigated. A fourth multiple linear regression model (Model 4) evaluated the interaction between lifetime psychedelic use and family history of psychotic or bipolar disorders on psychotic symptoms. The purpose of the fourth model was to determine whether psychedelic use might be more strongly associated with psychotic symptoms among those genetically predisposed to psychotic or bipolar disorders. As sensitivity analyses, we also ran all four analyses using multiple logistic regression models with the dependent variables dichotomized (i.e., one or more unusual visual experiences = 1, no unusual visual experiences = 0; one or more psychotic symptoms = 1, no psychotic symptoms = 0).

In all models, we controlled for broadly the same covariates that were used in the only prior study that has evaluated the associations between lifetime psychedelic use, unusual visual experiences, and psychotic symptoms in a sample representative of the US adult population (Krebs & Johansen, 2013): age in years, gender, ethnoracial identity, educational attainment, annual household income, marital status, engagement in risky behavior, and lifetime use of cocaine, sedatives, pain relievers, marijuana, phencyclidine (PCP), 3,4-methylenedioxymethamphetamine (MDMA/ecstasy), and inhalants (each drug use variable entered as a separate covariate). Due to a data collection error, not all covariates in Krebs and Johansen (2013) were included in this study (e.g., lifetime exposure to an extremely stressful event). For all analyses, p-values are reported with 3 decimal places, allowing the reader to estimate any p-value corrections of the reader’s choosing.

***Results***

***Descriptive statistics and sample characteristics***

Tables 1 and 2 show descriptive statistics and sample characteristics. As seen in Table 1, respondents who reported lifetime psychedelic use had significantly higher scores on unusual visual experiences across the lifetime than those who did not report previous psychedelic use, but there were no differences in past two-week psychotic symptoms between those who reported lifetime psychedelic use and those who did not (see Supplemental Table 1 for additional descriptive statistics). As shown in Table 2, personal and family histories of psychotic or bipolar disorders were significantly more common among psychedelic users. Notably, having been told by a doctor or other medical professional that they had HPPD was significantly more common among psychedelic users (1.3% versus 0.3% among non-users), but no differences were observed across groups in frequency or treatment of unusual visual experiences.

***Covariate-Adjusted Regression Models***

Table 3 presents results from four separate multiple linear regression models on the associations of lifetime psychedelic use with unusual visual experiences and psychotic symptoms. While lifetime psychedelic use was associated with more unusual visual experiences at any point across the lifetime, no association was observed between lifetime psychedelic use and recent psychotic symptoms. There was an interaction between lifetime psychedelic use and family (but not personal) history of psychotic or bipolar disorders on psychotic symptoms such that psychotic symptoms were highest​ among respondents who reported lifetime psychedelic use and a family history of psychotic or bipolar disorders, and lowest​ among those who reported lifetime psychedelic use and no family history of psychotic or bipolar disorders (see Supplemental Table 2 for adjusted means). Sensitivity analyses showed broadly the same results (see Supplemental Table 3 for results).

**Discussion**

The present study investigated the associations between lifetime naturalistic psychedelic use, lifetime unusual visual experiences, and past two-week psychotic symptoms in a sample representative of the US adult population with regard to sex, age, and ethnicity. Although the results in this study should be interpreted with caution, the findings suggest that lifetime use of psychedelics might be associated with more unusual visual experiences across the lifetime, as well as more psychotic symptoms in the past two weeks for individuals with a family history of psychotic or bipolar disorders and the reverse for those without such a family history.

Lifetime psychedelic use was associated with unusual visual experiences at any point across the lifetime. While such experiences may not have been functionally impairing, the results showed that 1.3 percent of respondents who reported lifetime psychedelic use had been told by a doctor or other medical professional that they had HPPD. Previous research on lifetime users of psychedelics and other substances (e.g., cannabis, ketamine, MDMA) found that at least 1.7 percent of respondents had a clear temporal relationship between drug use and onset of HPPD-like symptoms (Baggott et al., 2011), which is a statistic that broadly corresponds with the HPPD prevalence reported in this study. Given that potential risk factors (e.g., genetic, extra-pharmacological) of these types of experiences are not yet well-understood, future studies should use longitudinal research designs to better understand if, for whom, and under what circumstances psychedelic use might lead to unusual visual experiences.

Lifetime psychedelic use was not directly associated with recent psychotic symptoms, but there was an interaction between lifetime psychedelic use and family history of psychotic or bipolar disorders on psychotic symptoms such that psychotic symptoms were highest​ among respondents who reported lifetime psychedelic use and a family history of psychotic or bipolar disorders, and lowest​ among those who reported lifetime psychedelic use and no family history of psychotic or bipolar disorders. These findings suggest that there may be a genetic predisposition that puts certain individuals at risk of psychotic symptoms following use of psychedelics, which corresponds with the leading guidelines for psychedelic research (Johnson et al., 2008). Previous research suggests that psychotic disorders such as schizophrenia are genetically related to bipolar disorders (Ruderfer et al., 2018), but it is possible that the effects of psychedelic use on psychotic symptoms might differ between these disorders. It is also possible that psychedelics can induce a manic switch and put certain individuals at risk of mania (potentially with psychotic features; Gard et al., 2021), which highlights the need for future research to investigate psychotic and bipolar disorders separately and also to examine both psychotic and manic symptoms.

There are many features of the study design that should be considered when interpreting the results. First, causality cannot be inferred due to the cross-sectional research design. Any findings suggesting psychedelic use elicits unusual visual experiences or psychotic symptoms could also be interpreted to mean that unusual visual experiences or psychotic symptoms elicit psychedelic use. Consistent with this interpretation, family history of psychotic or bipolar disorders (i.e., a variable that could not be affected by lifetime psychedelic use) was more commonly reported among those who reported lifetime use of psychedelics. Second, even though the sample was stratified across sex, age and ethnicity to reflect the demographic distribution of the US adult population, it may not have been representative on other relevant variables such as income or educational attainment. It is also possible that associations in the covariate-adjusted models were influenced by other variables not included in the survey that are relevant to unusual visual experiences and psychotic symptoms (e.g., amphetamine use). Fourth, the survey item related to HPPD did not specify whether it concerned HPPD type I or II. Given that the estimated prevalence rate of HPPD type II among hallucinogen users is extremely low (Halpern et al., 2018), it is possible that those respondents who reported HPPD diagnosis by a doctor or other medical professional had received a HPPD type I diagnosis. It should be noted, however, that these may have been inaccurate diagnoses. For example, it is possible that distressing symptoms associated with psychedelic use were labeled HPPD as a diagnostic category necessary for third party reimbursement. Fifth, the questionnaires in this study used the same phrases that were used in the original questionnaires, which resulted in different time frames for unusual visual experiences (at any point across the lifetime) and psychotic symptoms (past two weeks). This suggests that the reported associations of psychedelic use with unusual visual experiences and psychotic symptoms may not be comparable. Sixth, respondents were asked whether they had a current or past history of a psychotic or bipolar disorder and also whether they had a first- or second-degree relative with a psychotic or bipolar disorder, which corresponds with criteria that would typically lead to exclusion from participation in a clinical trial with psychedelics. The respondents were not, however, asked to specify whether they had either a personal or family history of either psychotic disorders or bipolar disorders per se (or subtypes thereof), which would have been useful in determining the associations of each unique disorder. Future research should use longitudinal research designs to investigate interaction effects between psychedelic use and potentially relevant psychiatric histories (e.g., anxiety disorders) on unusual visual experiences. It would also be important to investigate interaction effects between psychedelic use and more specific psychiatric histories (e.g., schizophrenia, brief psychotic disorder, bipolar I disorder, bipolar II disorder) on psychotic and manic symptoms.

**Conclusions**

Despite the limitations of this study, the current results suggest that lifetime naturalistic use of psychedelics might be associated with more unusual visual experiences across the lifetime, as well as more psychotic symptoms in the past two weeks for individuals with a family history of psychotic or bipolar disorders and the reverse for those without such a family history. Future research should distinguish between different psychotic and bipolar disorders and specify subtypes of these mental health conditions, and should also utilize other research designs (e.g., longitudinal) and variables (e.g., polygenic risk scores) to better understand potential cause-and-effect relationships.

**References**

American Psychiatric Association (2013) Diagnostic and statistical manual of mental diseases (DSM-V), 5th edn. American Psychiatric Association Press, Washington, DC.

Andersen, K. A., Carhart‐Harris, R., Nutt, D. J., & Erritzoe, D. (2021). Therapeutic effects of classic serotonergic psychedelics: A systematic review of modern‐era clinical studies. Acta Psychiatrica Scandinavica, 143(2), 101-118.

Baggott, M. J., Coyle, J. R., Erowid, E., Erowid, F., & Robertson, L. C. (2011). Abnormal visual experiences in individuals with histories of hallucinogen use: A web-based questionnaire. Drug and alcohol dependence, 114(1), 61-67.

Barrett, F. S., Bradstreet, M. P., Leoutsakos, J. M. S., Johnson, M. W., & Griffiths, R. R. (2016). The Challenging Experience Questionnaire: Characterization of challenging experiences with psilocybin mushrooms. Journal of Psychopharmacology, 30(12), 1279-1295.

Carhart-Harris, R., Giribaldi, B., Watts, R., Baker-Jones, M., Murphy-Beiner, A., Murphy, R., ... & Nutt, D. J. (2021). Trial of psilocybin versus escitalopram for depression. New England Journal of Medicine, 384(15), 1402-1411.

Faul, F., Erdfelder, E., Buchner, A., & Lang, A. G. (2009). Statistical power analyses using G* Power 3.1: Tests for correlation and regression analyses. Behavior research methods, 41(4), 1149-1160.

Galvão-Coelho, N. L., Marx, W., Gonzalez, M., Sinclair, J., de Manincor, M., Perkins, D., & Sarris, J. (2021). Classic serotonergic psychedelics for mood and depressive symptoms: a meta-analysis of mood disorder patients and healthy participants. Psychopharmacology, 238(2), 341-354.

Gard, D. E., Pleet, M. M., Bradley, E. R., Penn, A. D., Gallenstein, M. L., Riley, L. S., ... & Woolley, J. D. (2021). Evaluating the risk of psilocybin for the treatment of bipolar depression: a review of the research literature and published case studies. Journal of Affective Disorders Reports, 6, 100240.

Goodwin, G. M., Aaronson, S. T., Alvarez, O., Arden, P. C., Baker, A., Bennett, J. C., ... & Malievskaia, E. (2022). Single-dose psilocybin for a treatment-resistant episode of major depression. New England Journal of Medicine, 387(18), 1637-1648.

Halpern, J. H., & Pope Jr, H. G. (2003). Hallucinogen persisting perception disorder: what do we know after 50 years?. Drug and alcohol dependence, 69(2), 109-119.

Halpern, J. H., Lerner, A. G., & Passie, T. (2018). A review of hallucinogen persisting perception disorder (HPPD) and an exploratory study of subjects claiming symptoms of HPPD. Behavioral neurobiology of psychedelic drugs, 333-360.

Johnson, M. W., Richards, W. A., & Griffiths, R. R. (2008). Human hallucinogen research: guidelines for safety. Journal of psychopharmacology, 22(6), 603-620.

Krebs, T. S., & Johansen, P. Ø. (2013). Psychedelics and mental health: a population study. PloS one, 8(8), e63972.

Kuzenko, N., Sareen, J., Beesdo‐Baum, K., Perkonigg, A., Höfler, M., Simm, J., ... & Wittchen, H. U. (2011). Associations between use of cocaine, amphetamines, or psychedelics and psychotic symptoms in a community sample. Acta psychiatrica scandinavica, 123(6), 466-474.

Lebedev, A. V., Acar, K., Garzón, B., Almeida, R., Råback, J., Åberg, A., ... & Petrovic, P. (2021). Psychedelic drug use and schizotypy in young adults. Scientific Reports, 11(1), 15058.

Müller, F., Kraus, E., Holze, F., Becker, A., Ley, L., Schmid, Y., ... & Borgwardt, S. (2022). Flashback phenomena after administration of LSD and psilocybin in controlled studies with healthy participants. Psychopharmacology, 1-11.

Roscoe, J., & Lozy, O. (2022). Can psilocybin be safely administered under medical supervision? A systematic review of adverse event reporting in clinical trials. Drug Science, Policy and Law, 8, 20503245221085222.

Ruderfer, D. M., Ripke, S., McQuillin, A., Boocock, J., Stahl, E. A., Pavlides, J. M. W., ... & Freedman, R. (2018). Genomic dissection of bipolar disorder and schizophrenia, including 28 subphenotypes. Cell, 173(7), 1705-1715.

Schlag, A. K., Aday, J., Salam, I., Neill, J. C., & Nutt, D. J. (2022). Adverse effects of psychedelics: From anecdotes and misinformation to systematic science. Journal of Psychopharmacology, 36(3), 258-272.

Simonsson, O., Sexton, J. D., & Hendricks, P. S. (2021). Associations between lifetime classic psychedelic use and markers of physical health. Journal of Psychopharmacology, 35(4), 447-452.

Strassman, R. J. (1984). Adverse reactions to psychedelic drugs. A review of the literature. The Journal of nervous and mental disease, 172(10), 577-595.

von Rotz, R., Schindowski, E. M., Jungwirth, J., Schuldt, A., Rieser, N. M., Zahoranszky, K., ... & Vollenweider, F. X. (2023). Single-dose psilocybin-assisted therapy in major depressive disorder: A placebo-controlled, double-blind, randomised clinical trial. EClinicalMedicine, 56.

Zimmerman, M., & Mattia, J. I. (2001). A self-report scale to help make psychiatric diagnoses: the Psychiatric Diagnostic Screening Questionnaire. Archives of general psychiatry, 58(8), 787-794.

| Table 1. Descriptive statistics of psychotic symptoms and unusual visual experiences | | | |
| --- | --- | --- | --- |
|  | Users (n=613) | Non-users (n=2,209) |  |
|  | Mean (SD) | Mean (SD) | *p* |
| Unusual visual experiences | 1.82 (1.89) | 0.75 (1.51) | <.001 |
| Psychotic symptoms | 0.30 (0.81) | 0.33 (0.86) | .396 |
| Note: Mean = the mean number of unusual visual experiences or psychotic symptoms in the group; SD = Standard Deviation; t-tests were used to compare the means across groups (users, non-users). | | | |

| Table 2. Sample characteristics of lifetime psychedelic users versus non-users | | | |
| --- | --- | --- | --- |
|  | Users  (n=613) | Non-users  (n=2,209) |  |
|  | n (%) | n (%) | *p* |
| 18 – 25 years old | 81 (13.1) | 379 (17.2) | .019 |
| Male | 332 (54.2) | 1,033 (46.8) | .001 |
| Non-Hispanic White | 523 (85.3) | 1,577 (71.4) | <.001 |
| Bachelor’s degree or higher | 301 (49.1) | 1,392 (63.0) | <.001 |
| Annual household income of ≥US$75,000 | 202 (33.0) | 900 (40.7) | <.001 |
| Single or never married | 172 (28.1) | 616 (27.9) | .933 |
| Never risk-taking | 47 (7.7) | 293 (13.3) | <.001 |
| Lifetime cocaine use | 425 (69.3) | 226 (10.2) | <.001 |
| Lifetime sedative use | 323 (52.7) | 518 (23.5) | <.001 |
| Lifetime pain reliever use | 510 (83.2) | 1,357 (61.4) | <.001 |
| Lifetime marijuana use | 595 (97.1) | 1,208 (54.7) | <.001 |
| Lifetime PCP use | 87 (14.2) | 31 (1.4) | <.001 |
| Lifetime MDMA/ecstasy use | 251 (41.0) | 102 (4.6) | <.001 |
| Lifetime inhalants | 244 (39.8) | 295 (13.4) | <.001 |
| Personal history of psychotic or bipolar disorders | 72 (11.8) | 83 (3.8) | <.001 |
| Family history of psychotic or bipolar disorders | 190 (31.0) | 411 (18.6) | <.001 |
| HPPD reported by doctor or other medical professional | 8 (1.3) | 6 (0.3) | .001 |
|  |  |  |  |
|  | Users (n=205) | Non-users (n=530) |  |
|  |  |  |  |
| Frequency of unusual visual experiences |  |  | .772 |
| Very rarely | 57 (27.8) | 153 (28.9) |  |
| Rarely | 62 (30.2) | 171 (32.3) |  |
| Occasionally | 62 (30.2) | 159 (30.0) |  |
| Very frequently | 21 (10.2) | 43 (8.1) |  |
| Constantly | 3 (1.5) | 4 (0.8) |  |
|  |  |  |  |
| Treatment of unusual visual experiences |  |  | .194 |
| Treatment not considered | 156 (76.1) | 369 (69.6) |  |
| Treatment considered | 29 (14.2) | 102 (19.3) |  |
| Treatment sought | 20 (9.8) | 59 (11.1) |  |
| Note: n refers to the number of respondents in the respective columns (users, non-users) who endorsed each item; percentages are reported within brackets. Respondents who endorsed any of the first seven listed unusual visual experiences (users, n = 205; non-users, n = 530) were asked about the frequency and treatment of those experiences. All percentages were rounded to the nearest 0.1%; cumulative percentages may not add to 100.0. Pearson's chi-squared tests were used to examine the characteristics of lifetime psychedelic users versus non-users. | | | |

| Table 3. Multiple linear regression model estimates | | | |
| --- | --- | --- | --- |
|  | B (CI 95%) | *p* | *R*^2^ |
| **Model 1** |  |  | .09 |
| Lifetime psychedelic use predicting lifetime unusual visual experiences | 0.31 (0.13 – 0.49) | .001 |  |
|  |  |  |  |
| **Model 2** |  |  | .13 |
| Lifetime psychedelic use predicting past two-week psychotic symptoms | -0.04 (-0.13 – 0.06) | .445 |  |
|  |  |  |  |
| **Model 3** |  |  | .13 |
| *Main effects predicting past two-week psychotic symptoms* |  |  |  |
| Lifetime psychedelic use | -0.05 (-0.15 – -0.05) | .318 |  |
| Personal history of psychotic or bipolar disorders | 0.39 (0.21 – 0.57) | <.001 |  |
| *Interaction effects predicting past two-week psychotic symptoms* |  |  |  |
| Lifetime psychedelic use X personal history of psychotic or bipolar disorders | -0.02 (-0.28 – 0.24) | .888 |  |
|  |  |  |  |
| **Model 4** |  |  | .13 |
| *Main effects predicting past two-week psychotic symptoms* |  |  |  |
| Lifetime psychedelic use | -0.10 (-0.21 – 0.00) | .052 |  |
| Family history of psychotic or bipolar disorders | 0.05 (-0.04 – 0.14) | .281 |  |
| *Interaction effects predicting past two-week psychotic symptoms* |  |  |  |
| Lifetime psychedelic use X family history of psychotic or bipolar disorders | 0.21 (0.05 – 0.38) | .010 |  |
| Note: B = Unstandardized beta; R^2^ = R-squared. Four separate multiple linear regression models were used to evaluate associations. All regression models controlled for age in years, gender, ethnoracial identity, educational attainment, annual household income, marital status, engagement in risky behavior, lifetime use of cocaine, sedatives, pain relievers, marijuana, phencyclidine (PCP), 3,4-methylenedioxymethamphetamine (MDMA/ecstasy), and inhalants. | | | |

| Supplemental Table 1. Additional descriptive statistics | | | |
| --- | --- | --- | --- |
|  | Users  (n=613) | Non-users (n=2,209) |  |
|  | n (%) | n (%) | *p* |
| Unusual visual experiences (≥1 of items below reported) | 244 (39.8) | 644 (29.2) | <.001 |
| Halos or auras around things | 100 (16.3) | 201 (9.1) | <.001 |
| Stationary things appear to move, breathe, grow, or shrink | 78 (12.7) | 165 (7.5) | <.001 |
| Things that are moving appear to be not moving | 41 (6.7) | 141 (6.4) | .785 |
| Things that are moving leave afterimages behind | 75 (12.2) | 155 (7.0) | <.001 |
| Colors increase in brightness or intensity | 116 (18.9) | 234 (10.6) | <.001 |
| You see with open eyes patterns or textures that are not really there | 96 (15.7) | 185 (8.4) | <.001 |
| You see with open eyes things or objects that are not really there | 35 (5.7) | 107 (4.8) | .386 |
| Oscillations or flashing light sources, as in TVs or fluorescent lights, bother you more than other times in your life | 132 (21.5) | 324 (14.7) | <.001 |
| Grids, gratings or closely spaced lines bother you more than other times in your life | 52 (8.5) | 155 (7.0) | .218 |
|  |  |  |  |
| Psychotic symptoms (≥1 of items below reported) | 102 (16.6) | 389 (17.6) | .575 |
| ...did things happen that you knew were true, but that other people told you were your imagination? | 60 (9.8) | 218 (9.9) | .953 |
| ...were you convinced that other people were watching you, talking about you, or spying on you? | 39 (6.4) | 185 (8.4) | .103 |
| ...did you think that you were in danger because someone was plotting to hurt you? | 22 (3.6) | 90 (4.1) | .586 |
| ...did you think that you had special powers other people didn’t have? | 25 (4.1) | 96 (4.4) | .772 |
| ...did you think that some outside force or power was controlling your body or mind? | 14 (2.3) | 65 (2.9) | .382 |
| ...did you hear voices that other people didn’t hear, or see things that other people didn’t see? | 23 (3.8) | 78 (3.5) | .794 |
| Note: n refers to the number of respondents in the respective columns (users, non-users) who endorsed each item; percentages are reported within brackets. All respondents (users, n = 613; non-users, n = 2,209) were asked about unusual visual experiences and psychotic symptoms. Pearson's chi-squared tests were used to examine the characteristics of lifetime psychedelic users versus non-users. | | | |

| Supplemental Table 2. Adjusted means from multiple linear regressions (Model 3 and 4) | | |
| --- | --- | --- |
|  | Users | Non-users |
|  | Adjusted mean (CI 95%) | Adjusted mean (CI 95%) |
| Personal history of psychotic or bipolar disorders | | |
| Yes | 0.63 (0.44 – 0.83) | 0.70 (0.53 – 0.88) |
| No | 0.26 (0.18 – 0.35) | 0.31 (0.28 – 0.35) |
| Family history of psychotic or bipolar disorders | | |
| Yes | 0.48 (0.36 – 0.61) | 0.37 (0.29 – 0.45) |
| No | 0.22 (0.13 – 0.31) | 0.32 (0.28 – 0.36) |
| Note: Adjusted mean = the adjusted mean number of psychotic symptoms in the group; CI 95% = 95% confidence interval. | | |

| Supplemental Table 3. Multiple logistic regression model estimates – Sensitivity analyses | | | |
| --- | --- | --- | --- |
|  | aOR (CI 95%) | *p* | *R*^2^ |
| **Model 1** |  |  | .06 |
| Lifetime psychedelic use predicting lifetime unusual visual experiences | 1.44 (1.11 – 1.86) | .005 |  |
|  |  |  |  |
| **Model 2** |  |  | .13 |
| Lifetime psychedelic use predicting past two-week psychotic symptoms | 0.89 (0.63 – 1.25) | .499 |  |
|  |  |  |  |
| **Model 3** |  |  | .14 |
| *Main effects predicting past two-week psychotic symptoms* |  |  |  |
| Lifetime psychedelic use | 0.81 (0.57 – 1.16) | .250 |  |
| Personal history of psychotic or bipolar disorders | 2.31 (1.36 – 3.93) | .002 |  |
| *Interaction effects predicting past two-week psychotic symptoms* |  |  |  |
| Lifetime psychedelic use X personal history of psychotic or bipolar disorders | 1.41 (0.64 – 3.10) | .392 |  |
|  |  |  |  |
| **Model 4** |  |  | .14 |
| *Main effects predicting past two-week psychotic symptoms* |  |  |  |
| Lifetime psychedelic use | 0.69 (0.46 – 1.03) | .066 |  |
| Family history of psychotic or bipolar disorders | 1.12 (0.82 – 1.53) | .482 |  |
| *Interaction effects predicting past two-week psychotic symptoms* |  |  |  |
| Lifetime psychedelic use X family history of psychotic or bipolar disorders | 1.97 (1.12 – 3.47) | .018 |  |
| Note: aOR = adjusted Odds Ratio; R^2^ = Pseudo-R-squared. Four separate multiple logistic regression models were used to evaluate associations. All regression models controlled for age in years, gender, ethnoracial identity, educational attainment, annual household income, marital status, engagement in risky behavior, lifetime use of cocaine, sedatives, pain relievers, marijuana, phencyclidine (PCP), 3,4-methylenedioxymethamphetamine (MDMA/ecstasy), and inhalants. | | | |
